# Immunogenicity and protective efficacy of an RSV G S177Q central conserved domain nanoparticle vaccine

**DOI:** 10.3389/fimmu.2023.1215323

**Published:** 2023-06-29

**Authors:** Harrison C. Bergeron, Jackelyn Murray, Maria G. Juarez, Samuel J. Nangle, Rebecca M. DuBois, Ralph A. Tripp

**Affiliations:** ^1^ Department of Infectious Diseases, College of Veterinary Medicine, University of Georgia, Athens, GA, United States; ^2^ Department of Biomolecular Engineering, University of California Santa Cruz, Santa Cruz, CA, United States

**Keywords:** RSV, G protein, structure-guided vaccine, nanoparticle vaccine, neutralizing Abs

## Abstract

**Introduction:**

Respiratory syncytial virus (RSV) can cause lower respiratory tract disease in infants and elderly populations. Despite decades of research, there remains no safe and approved RSV vaccine. Previously, we showed that an RSV G glycoprotein subunit vaccine candidate with a single point mutation within the central conserved domain (CCD), i.e. S177Q, considerably improved immunogenicity.

**Methods:**

Here, we examine the development of nanoparticle (NP) vaccines having either an RSV G protein CCD with wild-type sequence (NPWT) or an S177Q mutation (NP-S177Q). The NP vaccine immunogens were adjuvanted with monophosphoryl lipid A (MPLA), a TLR4 agonist to improve Th1- type responses. BALB/c mice were primed with 10 μg of NP-WT vaccine, NPS177Q, or vehicle, rested, and then boosted with a high (25 μg) or low (10 μg) dose of the NP-WT or NP-S177Q homologous candidate and subsequently challenged with RSV A2.

**Results:**

The results showed that mice boosted with NP-S177Q developed superior immunogenicity and neutralizing antibodies compared to NP-WT boosting. IgG from either NP-S177Q or NP-WT vaccinated mice did not interfere with fractalkine (CX3CL1) binding to CX3CR1 and effectively blocked G protein CX3C-CX3CR1 binding. Both NP-WT and NP-S177Q vaccination induced similar neutralizing antibodies to RSV in challenged mice compared to vehicle control. NP-S177Q boosting improved correlates of protection including reduced BAL cell infiltration following RSV challenge. However, the NP vaccine platform will require improvement due to the poor solubility and the unexpectedly weaker Th1-type IgG2a response.

**Discussion:**

The results from this study support further NP-S177Q vaccine candidate development.

## 1 Introduction

RSV is the leading cause of lower respiratory tract disease in infants and the elderly (1, 2). By age 2, nearly all infants have experienced RSV infection (3). RSV typically causes a mild upper respiratory tract infection, however severe respiratory disease presenting as bronchiolitis, pneumonia, and wheezing may require hospitalization (4). Infants <12 months of age are at the greatest risk for hospitalization (5). While preexisting conditions including preterm birth and cardiopulmonary abnormalities significantly increase susceptibility to RSV disease (6) previously healthy infants are also at risk for hospitalization (5, 7). Synagis^®^ (palivizumab) is an antibody against the RSV F protein that helps decrease the risk of serious lung infections and is restricted for use in at-risk infants (8). Its use in healthy infants is excluded thus countermeasures are currently unavailable in the United States (9–11). RSV infection may predispose infected infants to asthma and/or chronic wheezing later in life (12). Further, RSV infection does not induce robust antibody responses as reinfections are common (13). Maternal antibodies (Abs) provide protection against RSV, however, this protection wanes shortly after birth and the level of protection may vary (14, 15). Gaps remain in understanding the mechanisms of RSV disease, but it is understood that severe disease is linked to immunopathology (16). Thus, RSV vaccines that prevent immune-mediated pathology are needed to prevent severe RSV disease (17).

RSV has two major surface proteins, i.e. the F and G proteins. The F protein is indispensable for virus infection and is the antigen targeted by palivizumab and nirsevimab therapeutic antibodies (18, 19). While therapeutic anti-F protein antibodies (Abs) and serum anti-F protein Abs induced by RSV vaccine candidates are neutralizing and may provide some protection from disease (20), these Abs are insufficient at blocking RSV disease linked to the RSV G protein (17, 21–23). The RSV G protein is a heavily glycosylated surface protein comprised of three domains, i.e. the cytoplasmic (CT), transmembrane (TM), and ectodomain (ecto) domains. Importantly, the G protein ectodomain contains a central conserved domain (CCD) and CX3C motif that are highly conserved among RSV subtypes and strains (24). CX3C is the attachment motif for CX3CR1, or fractalkine receptor, that is expressed on human airway epithelial cells (hAECs) and some immune cells (25–30). G protein CX3C binding to CX3CR1 has been shown to induce aberrant CX3CR1+ T cell trafficking, modify host miRNA profiles, dampen antibody maturation, reduce antiviral cytokine and IFN responses, and potentiate Th2-type immune response during RSV infection (24, 31–37). Thus, the G protein affects RSV attachment and modifies host immune response to infection, and Abs that block the CX3C motif may prevent CX3C-mediated attachment and immune dysregulation (38, 39).

Anti-G protein Abs targeting the CCD and/or CX3C are protective, reduce Th2-type immune responses, increase antiviral IFN and T cell responses, and prevent lung pathology but the G protein itself is poorly immunogenic (40–43). The G protein has been implicated in vaccine-enhanced respiratory disease as early RSV vaccine trials with formalin-inactivated RSV (FI-RSV) resulted in vaccine-enhanced disease and two infant deaths following natural infection of FI-RSV vaccinees (44–46). Several studies have shown that G protein may prime for enhanced RSV disease (23, 32, 47, 48). Importantly, ablation of the CX3C motif to CX4C eliminates vaccine-enhanced disease showing that proper modifications to G protein can induce a protective response following vaccination while preventing disease (49, 50). Recently, we showed that the G protein with a single point mutation, i.e., S177Q, improved immunogenicity compared to wild-type G protein or CX4C G protein vaccination (51). A key finding was that the S177Q mutant, similar to CX4C, did not mediate CX3CR1+ immune cell trafficking illuminating how the S177Q mutant may resist the development of enhanced disease (52). Notably, unlike the CX4C mutant, the S177Q mutant was found to be structurally intact and display conformational epitopes for high-affinity anti-G Abs (52).

In this study, we made and evaluated nanoparticle (NP) immunogens displaying the CCD of the RSV G protein. We hypothesized that the self-assembling NPs would improve vaccine immunogenicity by presenting multiple copies of CCD antigens in a repetitive manner that is similar to natural infection. NPs displaying wild-type CCD (NP-WT), CCD containing the S177Q mutation (NP-S177Q), or no antigen (vehicle control) were used to immunize mice, followed by RSV challenge. NP-WT and NP-S177Q vaccine candidates were adjuvanted with MPLA to induce a Th1-type response (53, 54). Mice were intramuscularly (i.m.) primed with 10 µg of vehicle, NP-WT, or NP-S177Q vaccines and subsequently boosted with either 10 µg (low dose) or 25 µg (high dose) of the homologous NP vaccine candidates. Subsequently, mice were intranasally (i.n.) challenged with 10^6^ PFU RSV A2, and on day 5 post-challenge, lung viral loads and immune correlates were determined.

The results show the NP-S177Q vaccination induced greater immunogenicity compared to NP-WT or vehicle control. While both NP-WT and NP-S177Q vaccination reduced lung viral titers, NP-S177Q vaccination led to improved viral neutralization compared to NP-WT. IgG from NP-WT or NP-S177Q vaccinated mice did not interfere with FKN binding to CX3CR1, and the IgG blocked G protein binding to CX3CR1. Importantly, NP-S177Q vaccination was able to significantly reduce BAL cell infiltration following the RSV challenge compared to vehicle-vaccinated mice. This study shows that RSV G protein CCD nanoparticle vaccines have promise in the development of precision RSV vaccines, however as expected with novel vaccine development, will require optimization such as improving vaccine solubility. However, the findings of this study support improved NP platforms in developing the next generation of RSV G protein vaccines expressing the S177Q mutant.

## 2 Materials and methods

### 2.1 Cells and virus

Vero E6 (CRL-1586), A549 (CCL-185), HEp-2 (CCL-23), and HEK-293 (CRL-1573) (all from American Type Culture Collection (ATCC), Manassas, VA) were maintained in 10% fetal bovine serum (FBS)/DMEM (Hyclone, Logan, UT). CX3CR1.293 cells (>90% CX3CR1^+^) were maintained in selection media (10% FBS/DMEM + 1.0 µg/mL puromycin) as previously described (51). RSV A2 and B1 were propagated in HEp-2 cells as described (55). RSV A2 expressing green fluorescent protein (GFP) was propagated in HEp-2 cells as described (56).

### 2.2 Nanoparticle construction

Nanoparticle (NP) vaccines were constructed using self-assembling *Aquifex aeolicus* lumazine synthase (57) fused to a next-generation SpyCatcher domain (54, 58, 59). To generate the NPs, a pET28a plasmid encoding an N-terminal 6-histidine tag, the *Aquifex aeolicus* lumazine synthase protein (UniProtKB entry O66529), and SpyCatcher003 (58) (**Table 1**) was transformed into T7 Express *E. coli* and recombinant LumazineSynthase-SpyCatcher003 was expressed overnight at 18C. Cells were lysed by ultrasonication in wash buffer (10 mM Tris-Cl pH 8.0, 10 mM imidazole, 150 mM NaCl) with 1 mM MgCl_2_, protease inhibitors, benzonase, and DTT. *E.* coli lysates were clarified by centrifugation and 0.22 um filtered. LumazineSynthase-SpyCatcher003 was purified from clarified lysates by affinity chromatography using a HisPur Nickel-NTA Resin and eluted using wash buffer with 250 mM imidazole. LumazineSynthase-SpyCatcher003 was dialyzed into PBS (pH 7.4) overnight at 4°C, resulting in empty vehicle control NPs. For negative stain imaging, LumazineSynthase-SpyCatcher003 protein was deposited onto glow-discharged, carbon-coated 400 mesh copper grids, stained with 2% (w/v) uranyl-formate, and viewed on a 200 kV FEI Glacios transmission electron microscope.

**Table 1:** Nanoparticles.

**LS-NP (empty)**	*6xHis*-Lumazine Synthase Nanoparticle-SpyCatcher	MGSS*HHHHHH*SSGLVPRGSHMQIYEGKLTAEGLRFGIVASRFNHALVDRLVEGAIDCIVRHGGREEDITLVRVPGSWEIPVAAGELARKEDIDAVIAIGVLIRGATPHFDYIASEVSKGLANLSLELRKPITFGVITADTLEQAIERAGTKHGNKGWEAALSAIEMANLFKSLRSGGSGGGGMVTTLSGLSGEQGPSGDMTTEEDSATHIKFSKRDEDGRELAGATMELRDSSGKTISTWISDGHVKDFYLYPGKYTFVETAAPDGYEVATPIEFTVNEDGQVTVDGEATEGDAHT
**CCD-WT**	***SpyTag***-CCD-*6xHis-tag*	M ***RGVPHIVMVDAYKRYK*** GSKPNNDFHFEVFNFVPCSICSNNPTCWAICKRIPNKKPGKK *HHHHHH*
**CCD-S177Q**	>**SpyTag**-CCD-S177 ***Q***-*6xHis-tag*	M**RGVPHIVMVDAYKRYK**GSKPNNDFHFEVFNFVPCSIC ***Q*** NNPTCWAICKRIPNKKPGKK HHHHHH*

Nanoparticle construction of empty nanoparticle (LS-NP), CCD-WT (NP-WT), and CCD-S177Q (NP-S177Q); italicized = His-Tag, underline = lumazine synthase NP, CCD, or CCD-S117Q, capitalized normal = SpyCatcher003, bold = SpyTag003.

To generate CCD protein antigens, a synthetic gene encoding an N-terminal SpyTag003 (58) fused to RSV strain A2 G protein amino acids 157 to 197 (UniProtKB entry P03423) and a C-terminal 6-histidine tag was cloned into pRSFDuet-1 (**Table 1**). Recombinant SpyTag003-RSV G CCD WT and SpyTag003-RSV G CCD S177Q proteins were expressed in T7 Express *E. coli* overnight at 18C. Cells were lysed by ultrasonication in wash buffer (20 mM Tris-cl pH 8.0, 25 mM imidazole, 150 mM NaCl) with 1 mM MgCl_2_, protease inhibitors, and benzonase. *E. coli* lysates were clarified by centrifugation and 0.22 um filtered. SpyTag003-RSV G CCD WT and SpyTag003-RSV G CCD S177Q proteins were purified from clarified lysates by affinity chromatography using a HisTrap FF crude column and washed with wash buffer containing 6 M urea. Protein was eluted in wash buffer containing 500 mM imidazole. This CCD purification method has been used successfully to solve its structure bound to anti-G protein antibodies, confirming that the recombinant CCD protein adopts an antigenically-relevant conformation (52, 60, 61).

To generate CCD-coated NPs, LumazineSynthase-SpyCatcher003 protein was incubated with a 4 M excess of SpyTag003-RSV G CCD WT or SpyTag003-RSV G CCD S177Q overnight at 4°C. During this incubation, a covalent isopeptide bond is formed between the SpyTag003 and the SpyCatcher003 which is verified by SDS-PAGE and a shift in the molecular weight band. Aggregation was observed the following day and was confirmed to be the NP samples by SDS-PAGE. Insoluble pellets were resuspended in PBS (pH 7.4) to make a final concentration of 1 mg/ml. Protein concentrations for all NP samples were verified by Bradford assays.

### 2.3 Mice

Female BALB/c mice (10-to-12-weeks old; Jackson Laboratories, Bar Harbor, ME) were housed in micro isolator cages with 12h light/dark cycle, and fed *ad libitum*. The mice received a priming dose of 10 µg NP-WT, NP-S177Q, or empty NPs. All vaccines were adjuvanted with 10 µg monophosphoryl Lipid A (MPLA; VacciGrade™ from *S. Minnesota* R595, InvivoGen, San Diego, CA), a TLR4 agonist, diluted in PBS. Similar to a related study that used using SpyCatcher multimerization of a SARS-CoV-2 spike vaccine candidate to induce a potent neutralizing antibody response at 21 days post-priming (62), vaccinated mice were boosted with either 10 µg or 25 µg of homologous vaccine or empty NPs and 10 µg MPLA diluted in PBS. Mice were i.m. vaccinated in the left and right and left quadriceps with 0.05 mL/quadriceps. Sera were collected on days 0, 14, 28, and 35 post-boosts. On day 21 post-boost, mice were i.n. anesthetized with Avertin (2, 2, 2-Tribromoethanol), and i.n. and challenged with 0.05 mL 10^6^ PFU RSV A2 diluted in PBS. Mice were monitored daily and euthanized on day 5 pi. Sera, BAL, lungs, and spleen were collected and stored on ice during organ processing for assays described below.

### 2.4 Serum ELISA

Sera were evaluated for anti-RSV IgG levels as described (51). Briefly, high-binding ELISA plates (Corning, Corning, NY) were coated with 5 µg/mL RSV A2 or B1 lysate overnight at 4°C. The next day, wells were washed 3x with KPL wash buffer (1x KPL in distilled water (diH_2_O) (SeraCare, Milford, MA) and blocked with Blotto (5% non-fat dry milk) overnight at 4°C. Blotto was removed and sera (in 3-fold dilutions starting at 1:50) was diluted in Blotto and added to wells overnight at 4°C. Wells were washed 3x with KPL wash buffer and 2° goat-anti-mouse IgG-HRP (ThermoFisher, Waltham, MA), or secondary subtype IgG1 or IgG2a antibodies (Southern Biotech, Birmingham, AL) were added. Plates were incubated overnight at 4°C, washed 3x with KPL wash buffer, and developed with 1-Step™ Ultra 3,3’,5,5’-tetramethylbenzidine (TMB; ThermoFisher) for 20 min, and stopped with Stop Solution (ThermoFisher), then read immediately using a BioTek plate reader (BioTek, Winooski, VT) at OD_450_.

### 2.5 Microneutralization assay

To determine the level of RSV antibody neutralization in the mouse sera, a microneutralization assay was used as described with minor modifications (63). Briefly, sera were pooled and heat-inactivated at 55°C for 30 min. Diluted sera in 2% FBS/DMEM (1:40) were co-incubated with 200 FFU RSV A2-GFP +/- 10% guinea pig complement (C’) (Sigma-Aldrich, St. Louis, MO) for 1 h at 37°C. Following pre-incubation, the virus/sera mixture was added to 95% confluent A549 cells for 48 h. Fluorescent focus units (FFUs) were visualized using Cellomics ArrayScan (ThermoFisher), enumerated with HTS software, and mean FFUs of replicate wells were determined. Neutralization was determined as the percent reduction in mean FFUs compared to empty NP antisera.

### 2.6 CX3C-CX3CR1 blocking assay

A CX3C-CX3CR1 blocking assay was performed as described (24). Briefly, 500 nM RSV G ectodomain (G_ecto_) was incubated +/- 5/mL heparin sulfate (HS) (Sigma) to prevent non-specific binding and +/- 20 µg/mL IgG (isolated from vaccinated mice by Protein G beads (Invitrogen) for 1 h on ice. CX3CR1.293 and HEK-293 cells were harvested, and 4 x 10^6^ cells/mL were blocked with 1 µg/mL Fc block diluted in FACS buffer (0.8% FBS/PBS) for 15 min on ice followed by incubation with 500 nM RSV G_ecto_ +/- 5 µg/mL HS +/- 10 µg/mL IgG for 1 h on ice. Cells were washed and resuspended in 20 µg/mL anti-G protein mAb (clone 130-5F) for 45 min on ice. Cells were washed and resuspended in goat-anti-mouse Alexa488 (1:200) (ThermoFisher) for 45 min on ice and protected from light. Cells were washed 3x with FACS buffer, resuspended in FACS buffer, and analyzed by flow cytometry. To determine FKN blocking, the assay was followed similarly except cells were incubated with 2 µg/mL biotinylated-FKN (Acro Biosystems, Newark, DE) +/- 5 µg/mL HS and +/- 10 µg/mL IgG. To detect CX3CR1-bound FKN, cells were incubated with Streptavidin-PE (1:200) (ThermoFisher). Identical times and temperatures were used for both ligands. Percent inhibition was determined as the difference of CX3CR1^+^ binding (G or FKN +HS + vehicle IgG) – (G or FKN +HS +NP IgG or mAb control bound to) x 100 as previously described (51). At least 20,000 events were collected using BD LSR II (BD Bioscience, Franklin Lakes, NJ).

### 2.7 Plaque assays

Lungs were harvested at day 5 pi and homogenized in 1 mL DMEM using GentleMACS tissue homogenizer (Miltenyi Biotec, Gaithersburg, MD) as described (55). Homogenates were centrifuged at 500 xG at 4°C for 8 min, supernatant was 10-fold diluted in DMEM (Hyclone) and overlaid onto 90% confluent Vero E6 cells in 24-well plates. After 2h of absorption, cells were overlaid with 2% methylcellulose (Sigma Aldrich) and incubated at 37°C for 6 days. Following incubation, methylcellulose was aspirated, wells were washed with PBS, fixed with acetone: methanol (60:40, Sigma-Aldrich), and air-dried overnight. Wells were washed 3x with KPL wash buffer and blocked with blotto overnight at 4°C. The next day, Blotto was removed and a mAb cocktail against RSV F and G proteins (clones 131-2A, 131-2G) was diluted in blotto was added overnight at 4°C. Wells were washed 3x with KPL wash buffer and goat anti-mouse-AP (ThermoFisher) was added overnight at 4°C. Wells were washed 3x with KPL wash buffer and virus plaques were developed with 1-Step™ NBT/BCIP substrate solution (ThermoFisher) for 5 min, rinsed with diH2O, and enumerated using a dissection microscope.

### 2.8 BAL cell phenotyping

Bronchioalveolar leucocytes (BAL) were collected by i.p. anesthetizing (Avertin) mice and terminally bleeding by severing the left axillary artery. The trachea was exposed and a small incision was made. The lungs were flushed 3x with 1 mL PBS and collected in 1.5 mL snap-cap tubes and BAL was centrifuged for 10 min at 500 xG at 4°C. The supernatant (BAL fluid) was separated and stored at -80°C until cytokine/chemokine analysis. BAL cells were resuspended in FACS buffer (0.8% FBS/PBS) and enumerated using a hemocytometer and Trypan blue. Cells were washed with FACS buffer and resuspended in Fc Block for 15 min on ice followed by the addition of anti-CD3, anti-CD8, and anti-CD11b, or isotype control Abs (all from BD Bioscience) for 1h on ice (**Supplementary Figure 2**). Cells were washed, fixed with 2% PFA (Ted Pella, Redding, CA) for 20 min at room temperature, washed, and resuspended with FACS buffer. At least 10,000 events were collected with BD LSR II (BD).

### 2.9 Intracellular cytokine staining

Spleens from mice were collected at day 5 pi. Single-cell suspensions of spleen cells were made by dissociation through a 100 uM cell strainer (Corning), washed with Hanks Balanced Salt Solution (HBSS) (HyClone), and red blood cells were lysed with Gey’s solution (Sigma-Aldrich) for 5 min. Splenocytes were washed 2x with HBSS, resuspended in media containing 10% FBS + RPMI-1640, and enumerated using a hemocytometer, and 2 x 10^7^ cells/mL were plated in a round bottom 96-well plate (Corning). Splenocytes were stimulated with 10 µg/mL RSV G (_183_WAICKRIPNKKPGKK_197_) and M2 peptides (_82_SYIGSINNI_90_) (42) or control (GFP, aa 200-208), phorbol 12-myristate 13-acetate (PMA)/ionomycin (Sigma), or left unstimulated and were treated with GolgiPlug (Brefeldin A) (BD) to retain cytokines and incubated at 37°C for 6h. After 6 h, cells were washed 3x with FACS buffer, blocked with 1 µg/mL Fc block (BD), and stained with anti-CD3 and anti-CD4 or isotype controls (all from BD Bioscience) for 1 h on ice. Cells were fixed with 2% PFA for 20 min at room temperature, washed with permeabilization buffer (BD Bioscience), and incubated with anti-IFNγ and anti-IL-4 or isotype controls diluted in permeabilization buffer for 1 h at 4°C (**Supplementary Figure 2**). Cells were washed 3X with permeabilization buffer, resuspended in FACS buffer, and analyzed with BD LSR II (BD Bioscience) with at least 10,000 events collected.

### 2.10 Statistics

Data were analyzed by one-way ANOVA with Dunnett’s multiple comparison test. p<0.05 was considered significant. Data are represented as mean +/- SEM. A vaccination study was performed once. Experiments were performed at least in duplicate with representative data shown.

## 3 Results

### 3.1 Nanoparticle vaccine constructs

**Figure 1:**
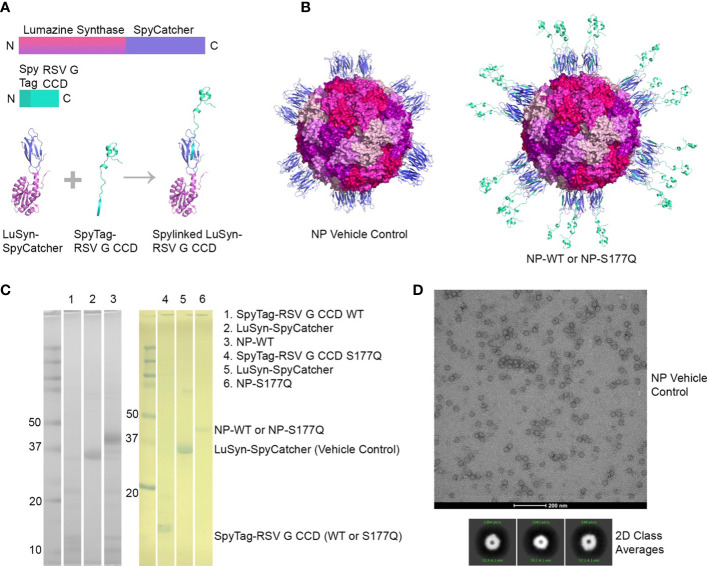
Production and characterization of RSV G CCD coated nanoparticle immunogens. **(A)** Schematic of lumazine synthase (LuSyn) (gradient purple/pink) and RSV G CCD (green cyan) expression constructs. SpyCatcher (periwinkle) is C-terminally fused to lumazine synthase. SpyTag (teal) is N-terminally fused to RSV G CCD constructs (WT or S177Q mutant). LuSyn-SpyCatcher and SpyTag-RSV G CCD are incubated together and are covalently linked *via* a spontaneous isopeptide bond formed between SpyTag and SpyCatcher proteins. **(B)** Representation of expected nanoparticle structures (prepared with PyMol version 2.5): Lumazine Synthase – SpyCatcher (empty NP, vehicle control) and Lumazine Synthase – RSV G CCD (NP-WT or NP-S177Q). 60 copies of lumazine synthase self-assemble to create 12 pentameric interfaces *via* their C-terminal ends thereby displaying 60 copies of spylinked RSV G CCD antigens. **(C)** SDS-PAGE of gel shift assay showing SpyTag - RSV G CCD (7.6 kDa), Lumazine Synthase-SpyCatcher (31 kDa), and NP-CCD WT or NP-CCD S177Q (38.6 kDa as a monomer) constructs after pelleting and resuspending in 1xPBS, pH 7.4. Multiple bands are likely due to contaminating proteins after Ni-NTA purification of bacterial lysates. **(D)** Negative stain electron microscopy micrographs (upper panel) and 2D class averages (lower panel) of empty NP’s show expected self-assembly and size.

NP immunogens were constructed using SpyTag/SpyCatcher technology (54, 58) (**Figure 1**). Briefly, a construct of lumazine synthase, which self-assembles into 60-mer spherical particles, was fused to a SpyCatcher domain. Recombinant lumazine synthase – SpyCatcher protein was purified and confirmed by negative stain electron microscopy to self-assemble into NPs (**Figure 1D**). To generate CCD-coated NPs, the lumazine synthase – SpyCatcher NPs were incubated with recombinant RSV G CCD protein fused to a SpyTag, allowing for the formation of the covalent isopeptide bond between the SpyCatcher and SpyTag and display of the CCD antigen on the surface of the NPs (**Figure 1B**). Covalent linking of the SpyTagged CCD to the lumazine synthase – SpyCatcher was verified by SDS-PAGE and a change in molecular weight of the bands (**Figure 1C**). We previously identified that a point mutation at site 177 (serine to glutamine) improved immunogenicity in a G glycoprotein vaccine compared to wild-type G protein adjuvanted with MPLA (51). Thus, in addition to wild-type CCD antigen loaded onto NPs (NP-WT), the S177Q CCD antigen was also generated and loaded onto NPs (NP-S177Q). Notably, upon overnight incubation of CCD antigens with NPs, precipitation was observed. Pelleting of the precipitate by centrifugation and evaluation by SDS-PAGE revealed that the precipitate is the NP-WT and NP-S177Q nanoparticle samples (**Figure 1C**). No precipitation is observed by incubation of empty NPs or CCD alone, suggesting that the loading of the CCD, which contains many hydrophobic amino acids, promoted insolubility of the NPs. To generate samples for immunization, pellets were resuspended in PBS.

### 3.2 RSV NP vaccines induce Anti-RSV Abs

**Figure 2:**
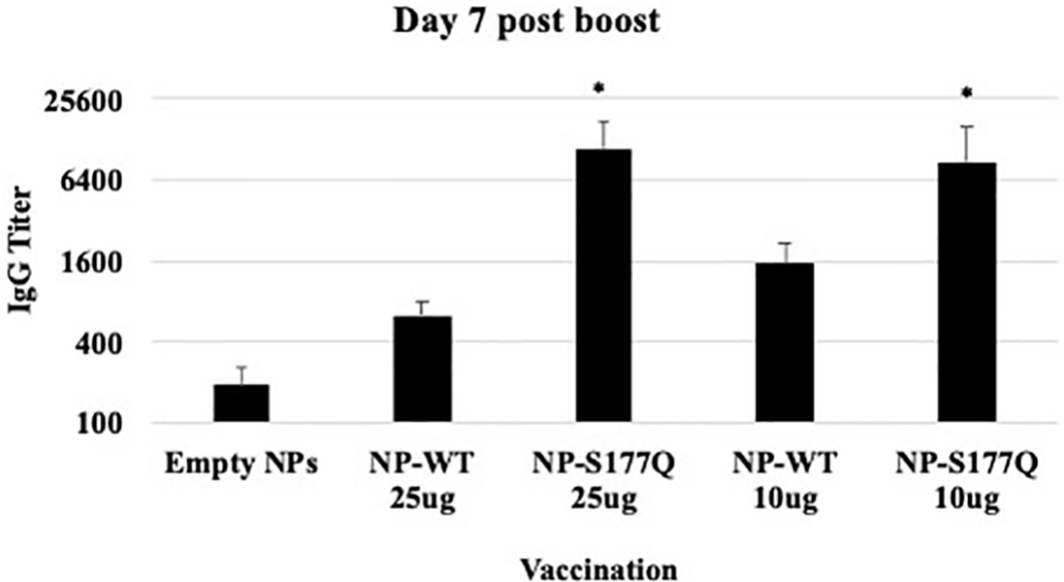
RSV G protein NP Vaccine Immunogenicity. Mice received a priming dose of 10 µg NP-WT, NP-S177Q, or empty NPs, all adjuvanted with 10 µg MPLA. On day 21 post prime, mice were boosted with either 10 µg or 25 µg of homologous vaccine or 10 µg empty NP, all adjuvanted with 10 µg MPLA. On day 7 post-boost, serum IgG responses were determined by ELISA. IgG titer determined as the highest dilution OD_450_ value above background plus two standard deviations. Bars represent mean IgG titer + SEM (n = 5 mice/group). *p<0.05 by one-way ANOVA with Dunnett’s multiple comparison test compared to empty NPs.

**Figure 3:**
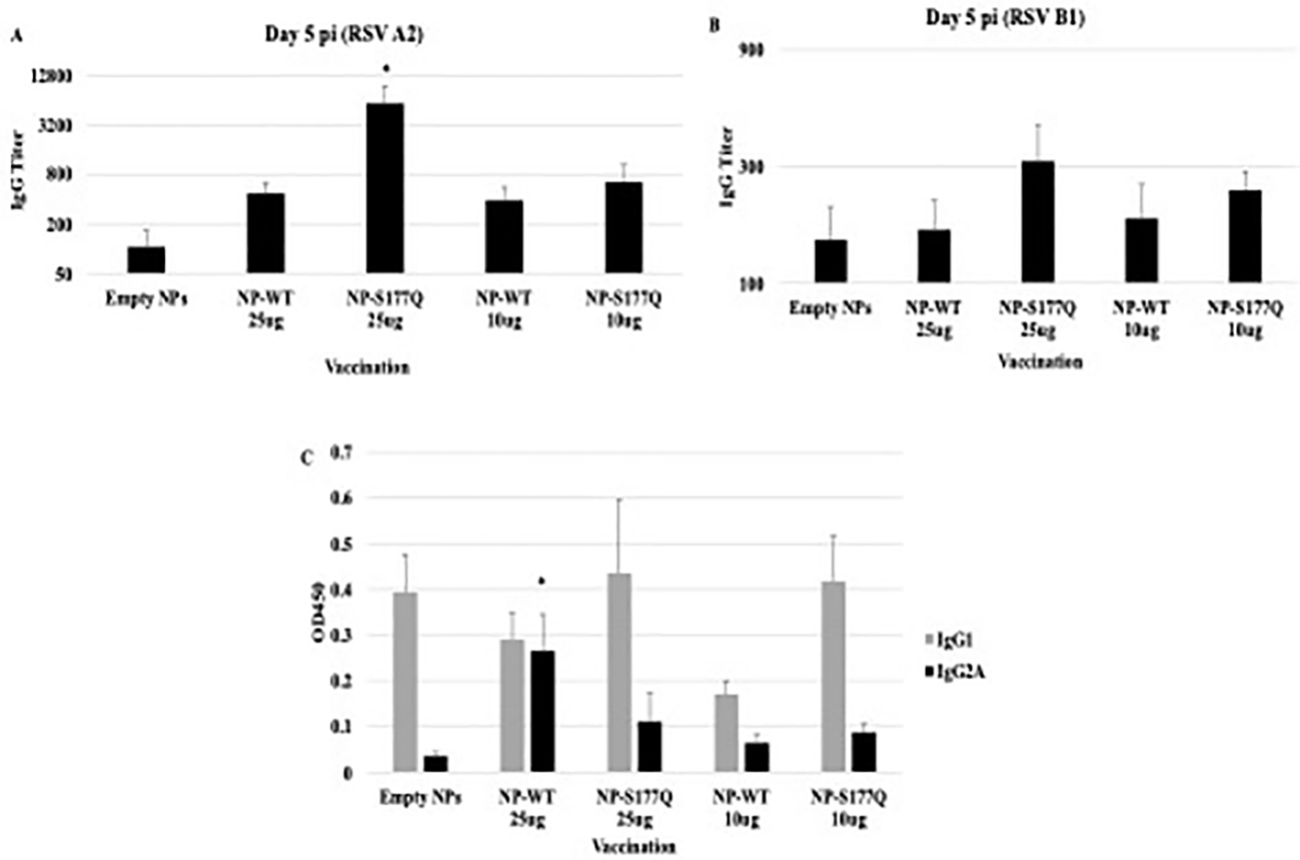
Serum Ab responses post-RSV challenge. Mice received a priming dose of 10 µg NP-WT, NP-S177Q, or empty NPs, all adjuvanted with 10 µg MPLA. On day 21 post prime, mice were boosted with either 10 µg or 25 µg of homologous vaccine or 10 µg empty NP, all adjuvanted with 10 µg MPLA. On day 21 post boost, mice were challenged with 10^6^ PFU RSV A2, and sera collected on day 5 pi. Ab responses were determined for **(A)** RSV A2 and **(B)** RSV B1. IgG titer determined as the highest dilution OD_450_ value above background plus two standard deviations. **(C)** OD_450_ values of IgG1 (gray) and IgG2A (black) responses against RSV A2. Bars represent mean IgG titer **(A, B)** or OD_450_ **(C)** +/- SEM (n=5 mice/group). *p<0.05 by one-way ANOVA with Dunnett’s multiple comparison test compared to empty NPs.

Mice received a priming dose of 10 µg NP-WT, NP-S177Q, or empty NPs adjuvanted with 10 µg MPLA. On day 21 post-prime, mice were boosted with either 10 µg or 25 µg of homologous vaccine or 10µg empty NP, all adjuvanted with 10 µg MPLA. At day 7 post-boost, the mice were bled, and anti-RSV Abs were detected by ELISA (**Figure 2**, **Supplementary Figure 1**). NP-WT and NP-S177Q vaccination induced anti-RSV Ab responses. Abs generated by NP-S177Q were significantly increased (p <0.05), and NP-WT 25µg and NP-WT 10µg Abs were increased (p = 0.28, p = 0.06, respectively) compared to empty NP vaccination. NP-S177Q vaccination induced moderately higher serum IgG titers than NP-WT (**Figure 2**), although the IgG responses did not statistically differ between vaccine doses. At day 21 post-boost, the NP-vaccinated mice were challenged with RSV A2, and on day 5 the serum Ab responses were determined. Similar to pre-challenge IgG titers, all vaccinated mice had greater anti-RSV A2 IgG compared to vehicle control (**Figure 3A**). Mice boosted with 25 µg of NP-S177Q vaccine had significantly (p<0.05) increased Ab titers compared to vehicle control, however, NP-S177Q vaccination was not statistically improved over NP-WT boosted mice. Contrary to our previous study demonstrating improved Ab recall responses (51), these data show a less robust recall response as sera Ab levels were roughly 1 log_3_ lower in each group on days 7 post boost and 5 post-challenge. Previous constructs utilized full-length G protein as opposed to restricting antibody responses to the CCD, which may partially explain this phenomenon. It is also possible Abs were present in the lung during infection and would not be detected in sera. It is also notable that serum Ab titers against RSV B1 were markedly lower than RSV A2 (**Figure 3B**). This finding was similar to a previous report suggesting anti-G Abs generated against A2 G protein bind with lower affinity to RSV B compared to RSV A2, likely due to variable residues encompassing the CCD between subtypes, despite conservation of the CX3C motif (40). NP-S177Q vaccination induced greater Abs compared to vehicle control. NP-WT also induced anti-B1 Abs although the titers were lower compared to NP-S177Q.

To determine if the serum Ab response were Th1- or Th2-like, ELISAs were performed to determine the specific IgG subclass (**Figure 3C**). It is established that IgG2a corresponds to a Th1-type response, while IgG1 corresponds to a Th2-type in response (64) and determines Fc effector function (e.g., complement-dependent cytotoxicity) (65, 66). There were no significant changes in Th2-type Ab responses between vehicle and NP-WT or NP-S177Q vaccinated mice. Further, Th1-type responses were only significantly (p<0.05) increased in the 25 µg NP-WT vaccinated mice, while there were no significant IgG2a responses in NP-S177Q vaccinated mice. These findings do not recapitulate the increased Th1-type responses which were previously observed with G protein immunogen (51). Conformationally designed epitopes such as those in the NP vaccines may require adjuvants that do not denature or emulsify the antigens, and or the insolubility of NP-WT and NP-S177Q vaccines may have contributed to these differences (67).

### 3.3 CX3C-CX3CR1 blocking

**Figure 4:**
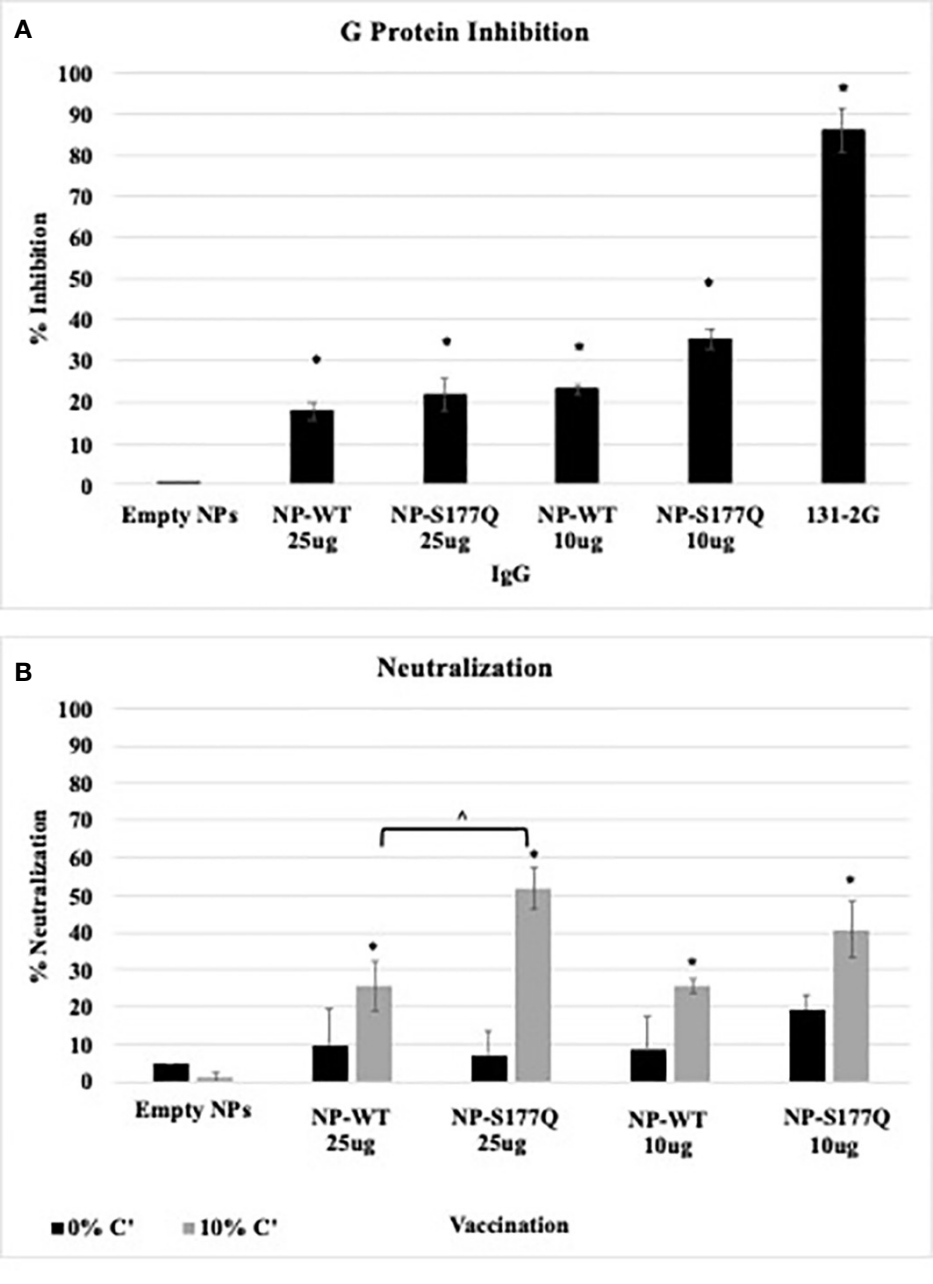
Ab Responses. Mice received a priming dose of 10 µg NP-WT, NP-S177Q, or empty NPs, all adjuvanted with 10 µg MPLA. On day 21 post prime, mice were boosted with either 10 µg or 25 µg of homologous vaccine or 10 µg empty NP, all adjuvanted with 10 µg MPLA. On day 21 post boost, mice were challenged with 10^6^ PFU RSV A2, and sera were collected on day 5 pi. **(A)** G protein CX3C-CX3CR1 blocking by IgG from challenged mice was determined by flow cytometry. **(B)** Pooled antisera were heat inactivated and diluted (1:40) for microneutralization assay in A549 cells with 0% (black) or 10% (grey) Guinea pig complement (C’). FFUs were collected on Cellomics ArrayScan and enumerated automatically with HTS Software (ThermoFisher). Bars represent mean + SEM (n=5 mice/group). **(A)** *p<0.05 by one-way ANOVA with Dunnett’s multiple comparison test compared to empty NP. For panel B, p <0.05 by one-way ANOVA with Tukey’s multiple comparison test to compare equally dosed NPs (^) and empty NPs (*).

Blocking CX3C-CX3CR1 interaction or ablating the CX3C motif is correlated with protection against RSV disease in mice and cotton rats (43, 49, 50, 68). To evaluate the efficacy of G protein CX3C-CX3CR1 blocking Abs generated in response to NP-WT or NP-S177Q vaccination, serum IgG from NP-vaccinated mice was isolated and tested. Similar to the G protein vaccinated mice (51), vaccination with NP-WT or NP-S177Q candidates induced significant (p<0.05) CX3C-CX3CR1 blocking Abs compared to vehicle IgG (**Figure 4A**), and Ab from NP-S177Q vaccination induced slightly higher blocking Abs (35%) than NP-WT vaccination (20%). As expected, mAb 131-2G which binds to a conserved epitope in the G protein blocked up to 90% G protein binding to CX3CR1. Contrary to our previous report showing that G protein induced greater CX3C-CX3CR1 blocking Abs compared to vaccination with an S177Q G protein mutant, in this study, we observed a slight improvement in G protein CX3C-CX3CR1 blocking, and in agreement with previous reports, 131-2G blocked G protein binding more effectively than polyclonal IgG from vaccinated mice. These Abs did not cross-react and block FKN binding to CX3CR1 (data not shown). This is not unexpected as there are structural differences that may preclude anti-G protein binding (69). Thus, this NP-S177Q vaccine platform induces G protein CX3C-CX3CR1 blocking Abs which have been shown to protect against RSV disease and are not implicated in modifying endogenous FKN signaling.

### 3.4 RSV neutralization

Anti-G protein Abs are neutralizing in human airway epithelial cells infected with RSV and *in vivo* (27). The addition of complement aids the neutralization of some anti-G protein Abs including the highly potent 3D3 and 3G12 anti-G protein mAbs that can be detected in immortalized cell lines (69, 70). To determine if serum from NP-WT or NP-S177Q vaccinated mice was neutralizing, heat-inactivated sera +/- 10% guinea pig complement (C’) were co-incubated with RSV-GFP (56) and added to RSV-infected human A549 cells for 48 h (**Figure 4B**). In the absence of complement, there was no significant (p>0.05) neutralization for any vaccine groups, however, serum plus complement from NP-WT and NP-S177Q vaccinated mice significantly (p<0.05) neutralized infected A549 cells compared to empty NP vaccination. Moreover, serum from 25 µg S177Q induced significantly (p<0.05) greater neutralization compared to NP-WT at the same dose. These data suggest neutralization is complement-dependent and not CX3C:CX3CR1-mediated neutralization.

**Figure 5:**
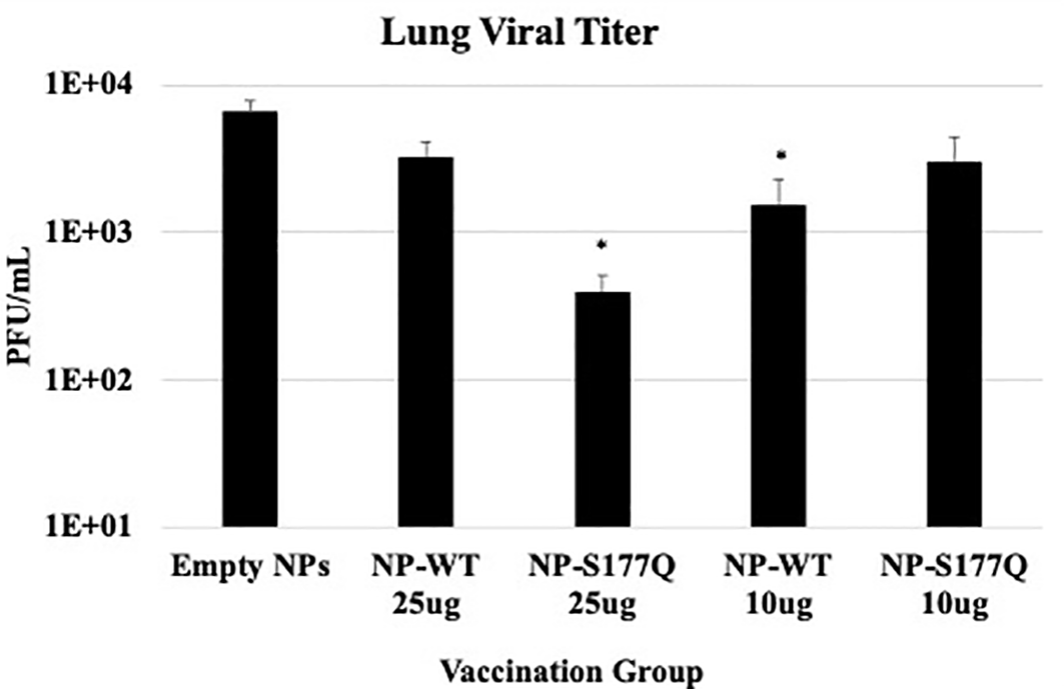
Lung Viral Titers. Mice received a priming dose of 10 µg NP-WT, NP-S177Q, or empty NPs, all adjuvanted with 10 µg MPLA. On day 21 post prime, mice were boosted with either 10 µg or 25 µg of homologous vaccine or 10 µg empty NP, all adjuvanted with 10 µg MPLA. On day 21 post boost, mice were challenged with 10^6^ PFU RSV A2, and at day 5 pi, lungs were harvested to determine virus loads. The bars represent the mean +/- SEM of plaque forming units (PFU)/mL of lung homogenate. *p<0.05 by one-way ANOVA with Dunnett’s multiple comparison test compared to empty NPs.

Lung viral titers showed that NP-WT and NP-S177Q vaccination reduces lung titers *in vivo* (**Figure 5**). On day 5 pi, corresponding to peak lung viral titers (71), 10 µg NP-WT or 25 µg NP-S177Q vaccination resulted in significantly (p<0.05) reduced viral titers in the lungs of RSV A2 challenged mice. 25 µg NP-WT vaccinated mice and 10 µg NP-S177Q vaccinated mice also reduced lung titers compared to empty NP vaccination (p=0.16, p=0.15, respectively). These findings are consistent with other G protein vaccines that reduce lung titers and induce anti-G protein neutralizing Abs (nAbs), however, others have reported that Abs to G protein are non-neutralizing but this was determined in the absence of complement, an effect which has caused misunderstanding (40, 41, 72, 73). There may be mechanisms aside from nAbs that result in reduced viral titers after NP vaccination including a cytotoxic T lymphocyte (CTL) response or improved macrophage activity, however these were not examined here. Moreover, while *in vivo* and *in vitro* neutralization data suggest 25 µg NP-S177Q vaccination resulted in the greatest levels of nAbs and reduced lung titers, the lack of significant *in vivo* reduction for 25 µg NP-WT and 10 µg NP-S177Q does not correlate with our findings *in vitro*. The findings from this study show that G protein immunogens are capable of inducing nAbs that are detectable *in vitro* with additional complement and vaccination may reduce lung viral titers in mice.

### 3.5 Immune response to RSV challenge

Aspects of RSV disease are connected with the expression of the G protein CX3C motif (50). Blocking G protein CX3C-CX3CR1 interaction with mAbs specific to this motif or the CCD domain is correlated with reduced RSV disease *in vivo* (40, 41, 73). To determine if NP-WT or NP-S177Q vaccination is protective against G protein-mediated disease, vaccinated mice were challenged with RSV A2 and BAL leukocytes were evaluated (**Table 2**). A significant (p<0.05) reduction in BAL cell numbers (3.9 x 10^4^ cells) in RSV-challenged mice that were vaccinated with 10 µg of NP-S177Q vaccine was evident compared to the empty NP control vaccinated mice (7.5 x 10^4^ cells). Interestingly, no other vaccination group including mice vaccinated with 25 µg of NP-S177Q vaccine had substantially reduced BAL cells following RSV challenge. Consistent with an overall reduction in BAL cell infiltration, RSV-challenged 10 µg NP-S177Q mice vaccinated had reduced CD11b+ cell numbers (2.3 x 10^3^) and a trend toward lower in CD8+ T cell numbers (2.0 x 10^3^) compared to RSV-challenged empty NP vaccinated mice (5.4 x 10^3^ and 3.3 x 10^3^ cells, respectively). Taken together, these support lung disease protection in mice vaccinated with NP-S177Q vaccine compared to vehicle control vaccinated mice. We also examined intracellular cytokine production by splenocytes stimulated with RSV G peptide encompassing the CCD and M2 as previously described (42, 68), however, there were no statistical differences detected in the production of IFNγ+ or IL-4+ by CD3+/CD4+ T cells between groups (data not shown).

**Table 2:** BAL Leukocytes.

	Empty NPs	NP-WT 25µg	NP-S177Q 25µg	NP-WT 10µg	NP-S177Q 10µg
**Total BAL Cells**	7.5 x 10^4^ *(± 4.7 x 10^3^)*	8.4 x 10^4^ *(± 12.0 x 10^3^)*	6.6 x 10^4^ *(± 14.0 x 10^3^)*	6.2 x 10^4^ *(± 6 x 10^3^)*	3.9 x 10^4^ * *(± 6.2 x 10^3^)*
**CD8+**	3.3 x 10^3^ *(± 5.5 x 10^2^)*	3.1 x 10^3^ *(± 4.3 x 10^2^)*	3.7 x 10^3^ *(± 8.2 x 10^2^)*	2.5 x 10^3^ *(± 5.2 x 10^2^)*	2.0 x 10^3^ *(± 1.0 x 10^2^)*
**CD11b+**	5.4 x 10^3^ *(± 6.0 x 10^2^)*	4.7 x 10^3^ *(± 6.8 x 10^2^)*	5.0 x 10^3^ *(± 17.0 x 10^2^)*	2.9 x 10^3^ *(± 4.8 x 10^2^)*	2.3 x 10^3^ *(± 2.4 x 10^2^)*

Mice received a priming dose of 10 µg NP-WT, NP-S177Q, or empty NPs, all adjuvanted with 10 µg MPLA. On day 21 post prime, mice were boosted with either 10 µg or 25 µg of homologous vaccine or 10 µg empty NP, all adjuvanted with 10 µg MPLA. On day 21 post boost, vaccinated mice were challenged with 10^6^ PFU RSV A2, and on day 5 pi the BAL cells were collected and enumerated (total BAL cells). CD8+ T cells and CD11b+ cells were determined by flow cytometry with at least 10,000 events collected. Mean total cells *±* SEM (shown in italics) are presented. *p<0.05 by one-way ANOVA with Dunnett’s multiple comparison test compared to empty NPs within the same row.

## 4 Discussion

RSV is a major cause of respiratory disease in the very young and old with no safe and approved vaccine available despite decades of research. The landscape of RSV vaccine research started with a failed formalin-inactivated RSV (FI-RSV) vaccine tested in the early 1960s (74). In those studies, FI-RSV vaccinated infants naturally infected with RSV infection resulted in a majority of infants requiring hospitalization where two infants died (44). Further investigation revealed that the FI-RSV vaccine caused enhanced disease (75). Moreover, it was later shown that Abs generated against RSV correlate with some but incomplete protection from disease, and that reinfection with identical RSV strains could occur, and that viral loads did not consistently correlate with disease severity in hospitalized infants (76–78). Thus, a safe and effective RSV vaccine has been elusive (20).

The RSV F protein has historically been the focus for RSV vaccine development as it is more conserved than G protein, and F protein is indispensable for *in vitro* infection (79). However, the G protein has a highly conserved CX3C chemokine mimic motif within its central conserved domain (CCD) (24, 60). Abs which bind the CCD and/or CX3C motif may be protective by preventing viral attachment to host cells as well as blocking G protein CX3C-CX3CR1 responses and G protein chemokine mimicry. Importantly, Abs induced by RSV G protein, including anti-G protein mAbs, that target the CCD and/or CX3C motif will neutralize RSV A and B strains, prevent Th2-type immune biasing due to G protein, reduce many of the immune correlates of severe RSV disease (e.g., eosinophilia), improve respiratory efforts, rescue protective IFN responses, and reduce lung pathology (17, 37, 80–85). At least two findings have stalled RSV G protein-based vaccine development, one being that the CCD region is poorly immunogenic compared to epitopes on F protein (86–88), and the G protein has been linked to the development of enhanced RSV disease (17, 22, 39).

To address these impediments, we have investigated the function and immunogenicity of various G protein mutants (49, 51, 68). Specifically, we examined the G protein S177Q mutant as a vaccine candidate because our studies showed that the mutation S177Q increased immunogenicity and improved Th1-type responses compared to G protein (51). The findings were predicted as immunogen was derived by structurally-guided vaccine development and knowing that a single point mutation in the CCD would alter the conformation of the G protein likely affecting its immunogenicity and safety profile. Structural and conformational validation showed that the CCD S177Q mutant retains high affinity when binding to mAbs and human anti-RSV reference sera and was substantially improved compared to the CX4C G protein mutant (52). In this study, mice were vaccinated with NP-WT or NP-S177Q generated with SpyTag/SpyCatcher technology (54, 58). Recently, a pre-F ferritin NP (pre-F-NP) with modified glycans was evaluated in mice and nonhuman primates (NHPs) (89). It was shown that pre-F-NP vaccination induced greater neutralizing antibody responses compared to DS-Cav1 trimer, suggesting the NP vaccine platform may offer superior characteristics compared to protein or subunit vaccination.

In this study, the NP-WT or NP-S177Q vaccine candidates were immunogenic in a prime/boost scheme, and consistent with our previous work, the NP-S117Q candidate showed improved immunogenicity. We sought to determine if these vaccines were protective, and to this end, the NP-vaccinated mice were i.n. challenged with RSV A2 and the serum antibody and BAL cell responses were determined. The sera responses after the RSV challenge were similar to the 25 µg NP-S177Q vaccinated mice, being significantly (p<0.05) more immunogenic than vehicle control, and NP-S177Q vaccinated mice also trended towards increased IgG titers compared to NP-WT for binding to RSV A2 and B1. As the CX3C motif is conserved between RSV subtypes and strains, these data suggest that Abs induced by NP-WT or NP-S177Q vaccination may be cross-reactive (40). The serum Ab isotypes were evaluated to determine if NP vaccination induced a Th1-dependent IgG2 response, or a Th2-dependent IgG1 response (64). Serum from NP-WT vaccinated mice indicated a predominantly Th1-type response, however, mice vaccinated with NP-S177Q predominantly had a Th2-type response (**Figure 3C**), which was inconsistent with our previous results. However, sera from both NP-WT and NP-S177Q vaccinated mice blocked G protein CX3C-CX3CR1 and did not interfere with FKN binding to CX3CR1 (**Figure 4**). Sera from 25 µg NP-S177Q vaccinated mice had significantly greater (p<0.05) complement-dependent neutralization activity in A549 cells compared to empty NP and 25 µg NP-WT. Thus, the Ab response to NP vaccination suggests NP-S177Q improves immunogenicity and induces greater nAbs, and that Abs that block G protein binding to CX3CR1.

Neutralizing the virus can contribute to reducing virus-mediated disease, however disease severity does not faithfully correlate with viral load or neutralizing Ab responses (77, 78, 80, 90–92). RSV disease is understood to be affected by both virus and host factors, and interventions that do not address G protein-mediated immune dysregulation may provide incomplete protection (22). While we noted significant reductions in lung viral loads in vaccinated mice, modalities that neutralize viruses and block G protein mediated disease are of great interest.

BAL cell influx during RSV infection is a correlate of immune-mediated disease (93). Initial vaccination with NP-WT did not prime for enhanced respiratory disease when the mice were boosted with NP-WT or NP-S117Q vaccines likely because of the MPLA adjuvant precluding non-neutralizing Th2-type responses and/or restriction of responses to the CCD. Mice receiving the 10 µg NP-S177Q vaccination resulted in significantly (p<0.05) fewer total BAL cells where CD11b+ and CD8+ BAL cells were substantially reduced while 25 µg vaccination did not have this result. It is possible that the 10 µg vaccine dose was suboptimal in terms of the robustness of BAL cell recruitment when the vaccinated mice were challenged. However, these findings show the NP-S177Q boosting effectively induces CX3C-CX3CR1 blocking and neutralizing Abs which can provide protection against RSV challenge and disease. Our previous study (51) evaluated various full-length mutant G proteins in a prime/boost/boost scheme, and we discovered significant Ab responses in mice vaccinated with S177Q mutations. Here, we describe the next iteration of this platform, an NP containing CCD with or without the S177Q mutation in a prime/boost scheme. Consistent with our previous studies, the NP-S177Q vaccine improves immunogenicity, however these studies do not demonstrate superiority to our previous full-length constructs. This may be due to the vaccination scheme (i.e., one versus two boosts), antigen delivery quality and/or presentation (e.g. poor solubility of NP constructs), or other differences. It will be important in future studies to compare various NP and microparticle (MP) vaccine platforms that improve solubility and immunogenicity and protect from disease. Our ongoing studies using these improved candidates will fully elucidate immune responses to this vaccine and show robust protection from disease.

## Data availability statement

The original contributions presented in the study are included in the article/**Supplementary Material**. Further inquiries can be directed to the corresponding author.

## Ethics statement

Mice studies were performed in compliance with all national and institutional guidelines and guidelines from the Human Care and Use of Laboratory Animals (American Association for Laboratory Animal Science) and performed according to a protocol approved by the University of Georgia Institutional Animal Care and Use Committee (IACUC) (A2022 04-023-Y1-A0, approval date 05/19/2022). All efforts were made to minimize animal pain and discomfort.

## Author contributions

HB designed and carried out experiments, wrote and edited the manuscript. JM data acquisition. MJ provided reagents. SN provided reagents. RD and RT secured funding and guided project, wrote and edited the manuscript. All authors contributed to the article and approved the submitted version.
