# Supplementary material for: Immunogenicity and protective efficacy of an RSV G S177Q central conserved domain nanoparticle vaccine
Source: Front Immunol. 2023 Jun 29;14:1215323. doi: 10.3389/fimmu.2023.1215323 (PMC10338877; doi:10.3389/fimmu.2023.1215323)
Supplement: Supplementary file 1 [file DataSheet_1.docx]

**Immunogenicity and Protective Efficacy of an RSV G S177Q Central Conserved Domain Nanoparticle Vaccine**

**Harrison C. Bergeron^1^, Jackelyn Murray^1^, Maria G. Juarez^2^, Samuel J. Nangle^2^, Rebecca M. DuBois^2^, and Ralph A. Tripp^1,*^**

^1^Department of Infectious Diseases, College of Veterinary Medicine, University of Georgia, Athens, GA

^2^ Department of Biomolecular Engineering, University of California Santa Cruz, Santa Cruz, CA

**Correspondence:**Ralph A. Tripp
[ratripp@uga.edu](mailto:ratripp@uga.edu)

**Supplementary Figure 1.** Sera ELISA from vaccinated mice against RSV A2 on day 7 post boost. Three-fold dilution of sera (starting at 1:50), reported as mean OD_450_ values (n = 5 mice/group)**.** Dashed line indicates limit of detection (LOD) = 0.13 OD_450_.

**Supplementary Figure 2. Flow Gating.** Flow gating strategy analyzed on FlowJo (V9) for (A) BAL cells. BAL cells were gated based on FSC/SSC for total cells or lymphocytes (FSC^lo^SSC^lo^). Total cells were further interrogated for CD11b+ or CD8+ cells.
